# Exploring facilitators and barriers to effective practice among new graduate occupational therapists working with children and families: A scoping review

**DOI:** 10.1111/1440-1630.70092

**Published:** 2026-04-27

**Authors:** Amanda Barnes, Laura Burritt, Laine B. Chilman, Pamela J. Meredith

**Affiliations:** ^1^ School of Health University of the Sunshine Coast Sippy Downs Queensland Australia; ^2^ Faculty of Health Sciences and Medicine Bond University Robina Queensland Australia; ^3^ Research Collaboration for Women and Children's Health and Sport Bond University Robina Queensland Australia; ^4^ Indigenous and Transcultural Research Centre University of the Sunshine Coast Sunshine Coast Queensland Australia; ^5^ School of Health and Rehabilitation Sciences The University of Queensland St Lucia Queensland Australia

**Keywords:** children, early career, NDIS, new graduate, occupational therapy, paediatric practice, scoping review, supervision, transition to practice, workforce development

## Abstract

**Introduction:**

Over the past three decades, occupational therapy literature has increasingly explored how new graduate occupational therapists transition into different areas of practice. However, limited evidence exists on the facilitators and barriers influencing new graduates' ability to work effectively in paediatric practice. In Australia, more new graduate occupational therapists are working with children and families, particularly in private settings that have expanded following the introduction of the National Disability Insurance Scheme (NDIS). In this context, understanding how new graduates develop competence is important for supporting effective practice in paediatric settings. The aim of this scoping review is to identify and synthesise the existing literature on the skills, attributes, facilitators, and barriers that influence new graduate occupational therapists' ability to work effectively with children and young people aged 0–18 years.

**Methods:**

This scoping review followed Preferred Reporting Items for Systematic Reviews and Meta‐Analyses extension for Scoping Reviews (PRISMA‐ScR) and Joanna Briggs Institute guidance. Databases were systematically searched for studies describing occupational therapists with less than three years of experience working with children and young people. One reviewer screened all titles, with independent screening of an initial sample by a second reviewer to ensure consistent interpretation of the eligibility criteria. Data were extracted using a piloted tool, and included studies were independently appraised for evidence level and quality. Findings were synthesised using conventional content analysis.

**Consumer and Community Involvement:**

This scoping review had no consumer or community involvement.

**Results:**

Of 3040 records identified, 13 met the inclusion criteria and were included in this scoping review. Four clusters were identified: (1) preparedness for the role; (2) setting or context matters; (3) support and continuing professional development; and (4) the new graduate experience.

**Conclusion:**

This scoping review provides a greater understanding of the experiences of new graduate occupational therapists working with children and families, and the workforce factors that enable or hinder effective practice and transition into paediatric roles. These findings are particularly relevant to contemporary Australian paediatric practice, where the introduction of the NDIS has influenced aspects of workforce and supervision structures. Findings also highlight priorities for education, supervision, and workforce planning to support new graduate occupational therapists working with children and families.

## PLAIN LANGUAGE SUMMARY

This scoping review explored what helps and what makes it difficult for newly qualified occupational therapists who work with children and young people aged 0–18. The first years of practice are known to be a time of learning and challenge, yet little is known about this experience in paediatric settings. To address this gap, research published over the past 30 years was searched, and 13 studies met the criteria for inclusion. The studies came from Australia, the United States, Canada, and New Zealand and used interviews and surveys to explore the views of newly qualified occupational therapists working in paediatrics. Across the studies, many new graduates felt underprepared for paediatric roles, particularly if they had limited clinical placement experience with children during their degree. Workplace context also played a major role, with factors such as caseload complexity, time pressures, service demands, and access to resources shaping their experiences. Support from supervisors and colleagues, opportunities for professional development, and structured learning helped new graduates build confidence, but these were not always available. The findings suggest that targeted university preparation, structured transition programs, and consistent supervision can support occupational therapists during their initial years working in paediatric practice. In Australia, the experiences of new graduates working with children and young people are influenced in part by the National Disability Insurance Scheme (NDIS), which has shaped how some paediatric services are delivered and the availability of supervision, mentoring, and professional support. A strength of this review is that it provides a clear summary of what is currently known and highlights areas for improvement across education, workplace support, and professional development. However, only a small number of studies were found, most used small samples, and few were conducted in Australia. Further high‐quality research is needed to inform future workforce strategies.

**Key Points for Occupational Therapy**

- New graduates face complex paediatric occupational therapy caseloads and often feel underprepared.
- This under preparedness may increase the risk of therapist burnout and challenges with workforce retention.
- Several supports can be activated to promote safe and effective service delivery.

## 1 INTRODUCTION

Occupational therapists work across the lifespan in diverse practice settings. The broad nature of the roles and responsibilities in the profession has made it challenging to determine how to best prepare graduates and what to expect of developing clinicians to support safe and effective practice (Toal‐Sullivan, 2006). Over the past three decades, national and international occupational therapy literature has explored factors influencing new graduates' experiences across different areas of practice such as aged care, mental health, acute and community adult care, vocational rehabilitation, and paediatric practice (Morley, 2009; Murray et al., 2020; Seah et al., 2011; Toal‐Sullivan, 2006; Turpin et al., 2021), reflecting a longstanding interest in the new graduate journey and how best to prepare clinicians to succeed in the early years of practice. Within this body of work, some studies have focussed on the educational needs of students preparing for specific areas of practice such as paediatrics, palliative care, and mental health (Crist et al., 2004), while others have explored the development of core skills such as communication and clinical reasoning (Gray et al., 2012; McCombie & Antanavage, 2017; Moir et al., 2021). For the purpose of this review, ‘area of practice’ refers to distinct domains within occupational therapy, such as paediatrics or mental health, while ‘practice setting’ refers to the environment in which services are delivered, such as hospitals or schools. Additionally, ‘new graduate’ refers to occupational therapists within the first three years following qualification working in paediatric practice. This operational definition was applied consistently when selecting studies and interpreting findings, as the term ‘new graduate’ is defined variably across the literature. Establishing this definition allows clarity when interpreting evidence about the transition from student to independent practitioner in paediatric practice.

Despite this growing body of research, paediatric practice is often not considered separately from other areas of practice (Gilman, 2011; Hummell & Koelmeyer, 1999; Moir et al., 2021; Morley, 2009; Murray et al., 2015; van Stormbroek & Buchanan, 2019). This lack of specific consideration within the literature is notable because the diversity of paediatric practice settings and client needs can influence new graduates' experiences, including the development of competence and access to professional support. In contemporary Australian practice, new graduates' experiences may also be affected by the National Disability Insurance Scheme (NDIS), which has shaped aspects of service delivery, including access to supervision and mentoring, and the time available for professional support (Jackson et al., 2023). In this context, understanding the facilitators and barriers that guide new graduates' development of competence is important for supporting effective practice in paediatric settings.

All occupational therapists are guided by the Australian Occupational Therapy Competency Standards (Occupational Therapy Board of Australia, 2018), which outline foundational capabilities expected across all practice areas. In paediatric practice, these standards are applied by adapting generalist competencies to meet the developmental, relational, and contextual needs of children, young people, and their families, while navigating complex systemic factors and collaborating across education, health, and community sectors (Murray et al., 2015). These demands require new graduates to apply their skills effectively within the multifaceted environments in which paediatric services are delivered. New graduates also engage in self‐directed learning and problem‐solving, proactively seeking knowledge, adapting approaches to individual children, and identifying gaps in their skills to support their ongoing development (Moir et al., 2021; Murray et al., 2020). This does not suggest that paediatric practice is inherently more difficult than other areas of practice, but rather that it presents a unique constellation of expectations and challenges that warrant targeted preparation and support.

Clinicians in paediatric practice often balance clinical knowledge, professional reasoning, family‐centred care, and collaboration with diverse stakeholders, including schools and community partners (Moir et al., 2021; Murray et al., 2015). While all occupational therapists graduate with foundational skills, it is increasingly acknowledged that certain practice areas may require additional skill development or targeted support during the early years of practice (Brandenburger‐Shasby, 2005). Documents such as the Occupational Therapy Australia Paediatrics Capability Framework (Occupational Therapy Australia, 2025b) aim to capture skill progression by providing a structured guide outlining the essential capabilities required for safe and effective paediatric practice at different levels of capability, across five key domains: (1) knowledge of paediatric systems, models, frameworks, and conditions; (2) guiding principles for working with families, caregivers, and significant others; (3) occupation‐focussed assessment and planning; (4) occupation‐focussed interventions and therapeutic strategies; and (5) working with other professionals and workforces. These domains represent a shared understanding of expectations for occupational therapists working with children and serve as a reference for universities, employers, and clinicians (OTA, 2025b). However, the operationalisation of this framework in practice settings has not been explored. Complementing this, the Essential Supports for Early Career occupational therapies (Occupational Therapy Australia, 2025a) highlights the importance of supervision, mentoring, structured learning, and peer connection, although the consistency of these supports in paediatric contexts remains unclear.

Given the varied nature of the existing evidence and the limited synthesis specific to paediatric practice, a scoping review is an appropriate method to summarise current knowledge and identify areas that may require further exploration (Peters et al., 2020). This scoping review aimed to map and synthesise the current literature on the skills, attributes, facilitators, and barriers experienced by new graduate occupational therapists working with children and young people aged 0–18 years.

## 2 METHODS

To address this aim, a scoping review was conducted to identify and synthesise the existing literature regarding facilitators and barriers to new graduate occupational therapists working with children and young people aged 0–18 years. A protocol for this scoping review was registered on the Open Science Framework (Barnes et al., 2024) to prevent duplication and encourage collaboration and transparency. To ensure rigour and accuracy in the process and outcomes, this review followed the five stages of the Joanna Briggs Institute methodology for scoping reviews (Peters et al., 2020): identifying the research question; identifying the relevant studies; selecting the studies; charting the data; and clustering, summarising, and reporting the results (Arksey & O'Malley, 2005). The Preferred Reporting Items for Systematic Reviews and Meta‐Analyses extension for Scoping Reviews (PRISMA‐ScR) checklist was also used to comprehensively answer the research question detailed below, provide a summary of the evidence, and identify key concerns and knowledge gaps (Tricco et al., 2018).

### 2.1 Stage 1: Identifying the research question

The research questions were developed using the Participants/Concepts/Context (PCC) framework (Peters et al., 2020) and are as follows:

1. What is currently known about the skills and attributes required of new graduate occupational therapists working with children and young people aged 0–18 years?
2. What facilitators and barriers have been reported that influence the ability of new graduate occupational therapists to work effectively in paediatric practice?

These questions guided all subsequent stages of the review, including study identification, selection according to eligibility criteria, data extraction, and synthesis of findings. Further details of the PCC framework used to develop these questions are provided in Table S1.

### 2.2 Stage 2: Identifying relevant studies

The search strategy was developed by the research team, with input from the health research librarian at The University of the Sunshine Coast. A three‐step search strategy was utilised in this review. First, an initial limited search of Scopus was undertaken to identify literature on the topic. The text words contained in the titles and abstracts of relevant articles were used to develop a full search strategy for Scopus, PubMed, and CINAHL (EBSCO). The search strategy, including all identified keywords, was adapted for each database, and systematic searches were conducted. The search terms included keywords and Boolean operators: graduate* OR unskilled OR novice OR ‘recent grad*’ OR ‘early career’ OR inexperienced OR ‘entry level’ AND ‘occupational therap*’. No language or date restrictions were applied, ensuring all relevant available literature was identified. Grey literature searching included reviewing professional occupational therapy association websites, from which two relevant documents were identified and included in the review.

Table 1 summarises the inclusion and exclusion criteria applied in this review. The primary inclusion criteria were (1) studies describing an occupational therapy caseload that included children and young people aged 0–18 years and (2) studies involving occupational therapists with less than 3 years of post‐graduate practice experience. In some studies, participants were described as ‘early career’ or ‘mentees’ without explicitly labelling them as ‘new graduates’. In these cases, studies were included if the majority of participants appeared to meet the less than 3 year criterion, even if a small proportion slightly exceeded this range. This approach allowed the inclusion of studies such as Thompson et al. (2024) and Jackson et al. (2023), which provided relevant insights into the experiences of early career therapists and mentees working with paediatric populations.

**TABLE 1:** Inclusion and exclusion criteria for a scoping review.

Inclusion	Exclusion
International literature	Describes professions other than occupational therapy.
Studies that include occupational therapists with less than 3 years post‐graduate experience.	Does not define or discuss new graduate role.
Contains a description of practice within the occupational therapy caseload.	Does not describe a specific type of service provision.
Describes an occupational therapy caseload which includes children 0–18 years of age.	Describes a caseload of people more than 18 years of age.
All publication types, including grey literature.	Describes clinicians with substantially more than 3 years of practice experience (e.g., mid or late career).
	Describes ‘graduate students’ such as Master's level graduate entry students, or undergraduate students.
	Focusses solely on university curriculum, without reporting post‐graduate clinical practice.

### 2.3 Stage 3: Study selection

A total of 3040 citations were identified from database searching, and 1188 duplicates were removed. The remaining 1858 citations were imported into Covidence for screening (Veritas Health Innovation, 2024). Level 1 screening involved a pilot test of the eligibility criteria on a random sample of 25 titles and abstracts by two reviewers (A. B. and L. B.). Discrepancies were discussed, and eligibility criteria were refined to achieve agreement. Level 2 screening involved one reviewer (A. B.) screening all 1858 titles and abstracts, while a second reviewer (L. B.) independently screened the first 1279 titles and abstracts to verify consistent application of the inclusion and exclusion criteria (Polanin et al., 2019). The remaining titles and abstracts were then screened by the first reviewer (A. B.), resulting in 175 papers retrieved for full‐text review. In addition to database searching, health service websites and publicly available documents were screened. These sources provided general guidance on competencies and support structures but did not report new graduates' experiences with paediatric‐specific caseloads, and therefore, no additional titles were included.

**FIGURE 1:**
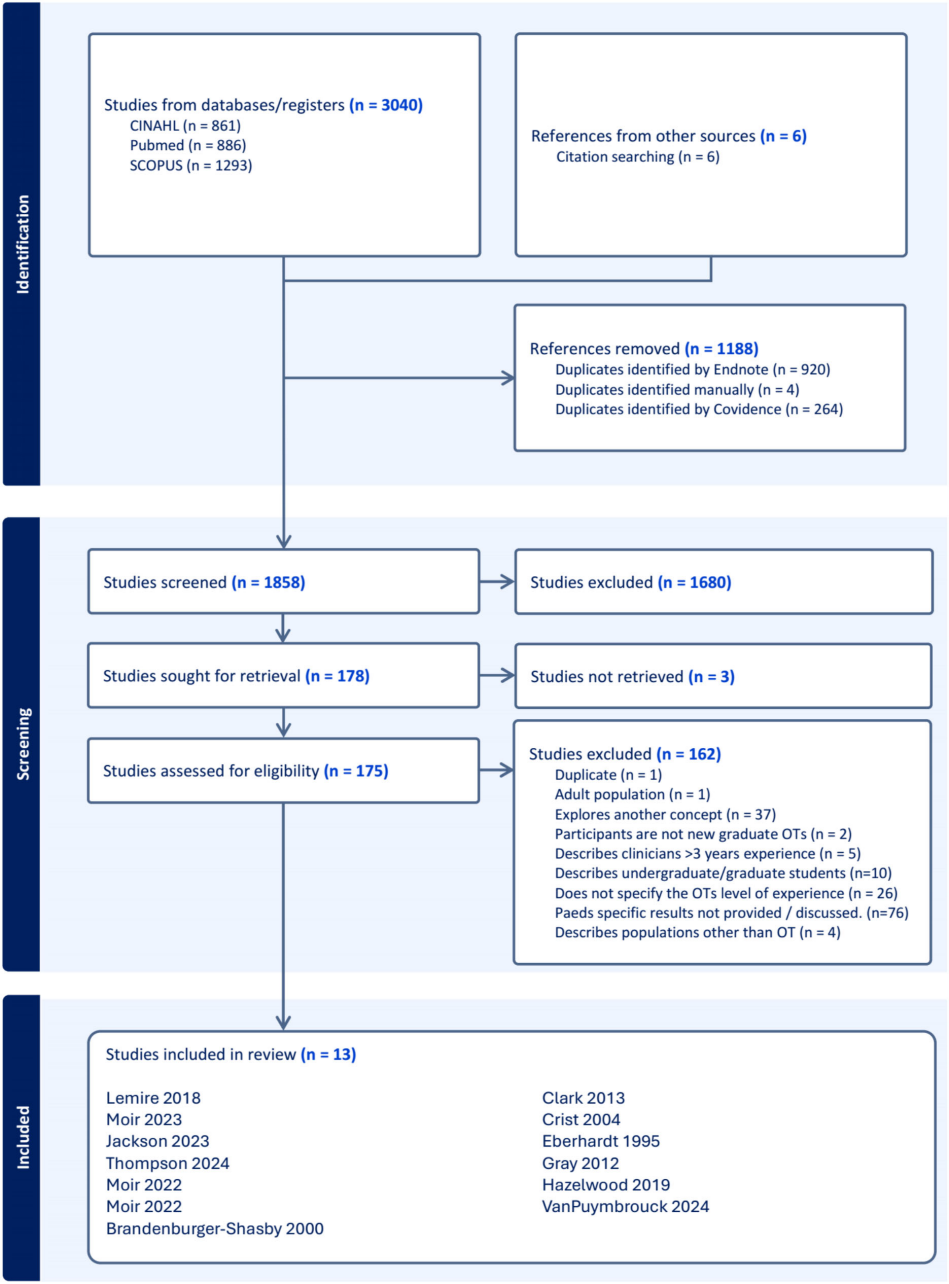
Preferred Reporting Items for Systematic Reviews and Meta‐Analyses (PRISMA) flow diagram for Study 1: Scoping review*. Note:* Adapted from Moher et al. (2009), PRISMA statement.

Following the Arksey and O'Malley (2005) framework, one reviewer (A. B.) screened all full texts, with a second reviewer (L. B.) independently checking the first 13 full texts to confirm that the inclusion/exclusion criteria were applied appropriately. Reasons for exclusion are documented in the PRISMA flow diagram (Figure 1), including ‘paediatric‐specific results not provided or discussed’, ‘does not specify the level of experience’ and ‘study explores another concept’. Three papers were unable to be sourced for full text review, with one paper unable to be sourced in English through available libraries. The authors of two papers were contacted via email to request clarification of the data to inform decisions regarding whether to include. One author was not able to recall study details, and the second author did not reply, resulting in the exclusion of both papers from the scoping review. An additional six titles sourced through handsearching reference lists were added to the full‐text review phase and were assessed for eligibility against the inclusion criteria. Following the screening process, a total of 13 studies were considered eligible for inclusion in this review.

### 2.4 Stage 4: Charting the data

Data were extracted from all 13 included papers using a structured extraction tool developed by the review team. The tool captured key information relevant to the review question, including participant characteristics, study aims, design, results, and key findings (summarised in Table 2). The tool was pilot tested on two studies, each independently extracted by two reviewers (A. B. and L. C.), followed by discussion to reach consensus and refine the tool. The first reviewer then completed extraction for all remaining included studies.

**TABLE 2:** Summary of included studies.

Author /year/country	Study aims	Study design/type	Evidence level	Participant experience	% of paed caseload	Participants <3 years/early career	Practice setting/area of practice
Brandenburger‐Shasby (2000), USA	To examine the perceptions of occupational therapies working in school‐based settings regarding their preparedness to practice based on pre‐service education.	Thesis.	Not applicable—thesis^a^	Mixed (NG < 3 years)	100%	141	Schools
Clark et al. (2013), Australia and New Zealand	To explore the perceptions of near‐misses and mistakes among new graduate occupational therapists from Australia and NZ, and their knowledge of current incident reporting systems.	Mixed methods, survey; journal article.	3B	<1 year	12.7%	228	Paediatrics (*n* = 29), mental health, adults, and other.
Crist et al. (2004), USA	1. To examine current entry‐point practice in the profession of occupational therapy. 2. To ensure that the knowledge, skills or abilities assessed in credentialing examinations are limited to those required in professional practice.	Quantitative, survey; journal article.	3B	<3 years	27%	1,003	Schools (*n* = 270), skilled nursing facility, acute care hospital, rehab hospital, outpatient community centre, and other.
Eberhardt and Mayberry (1995), USA	To explore entry‐level occupational therapists' attitudes towards persons with disabilities and their equal status and nonclinical contact with this population.	Quantitative, cross‐sectional survey; journal article.	3B	<2 years	10%	172	School (*n* = 16), general hospital, rehabilitation hospital‐centre, and skilled nursing facility.
Gray et al. (2012), Australia and New Zealand	To explore the feelings of newly graduated occupational therapists in Australia and Aotearoa/NZ regarding their education and work preparedness.	Qualitative, survey; journal article.	3B	<3 years	30%	231	Not stated.
Hazelwood et al. (2019), Australia	What are new graduate occupational therapists narratives of ethical tensions in practice?	Qualitative study using narrative enquiry; journal article.	3B	<1.5 years	25%	8	Public health paediatrics and subacute (*n* = 1), private practice (*n* = 1), mental health (*n* = 2), and workplace injuries (*n* = 1). Aged care (*n* = 3).
Jackson et al. (2023), Australia	1. To understand the role of mentorship in professional development support for novice occupational therapists working in community paediatric practice. 2. Examine the contribution of mentorship to the development of professional capability in paediatric occupational therapy practice from the perspective of mentors and mentees.	Qualitative; journal article	3B	Mixed (novice <5 years)	100%	8	Private practice (*n* = 5); non‐government organisation (*n* = 2); special school (*n* = 1).
Lemire (2018), Canada	Share individual perspective on new graduate experience.	Shared perspective; practice magazine.	N/A	<1 year	100%	1	Community (*n* = 1)
Moir et al. (2023), Australia	Explore new graduate occupational therapists experiences of learning to make intervention decisions in paediatric practice.	Qualitative, case study approach; journal article.	3A	Mixed (NG < 2 years)	100%	18	Private practice (*n* = 11): homes, clinics, and schools. Hospital (*n* = 4): inpatient and outpatient. NGO (*n* = 3).
Moir, Turpin, and Copley (2022), Australia	To explore new graduates' experiences of learning to make intervention decisions in paediatric private practice in Australia.	Qualitative, case study approach; journal article.	3A	<2 years	100%	11	Clinic‐based, school, and home visits.
Moir, Turpin, and Copley (2022), Australia	To describe how new graduates experience the process of learning to make intervention decisions within an Australian acute paediatric hospital setting.	Qualitative case study approach; journal article.	3A	Mixed (NG < 2 years)	100%	4	Paediatric hospital (*n* = 4).
Thompson et al. (2024), USA	1. Describe the education and training of SLPs and occupational therapists as it relates to assessment and treatment of PFD. 2. Determine the relationship between PFD education and training and clinicians' feelings of effectiveness in clinical practice.	Mixed methods, survey; journal article	3B	Mixed	100%	37	Hospital inpatient/outpatient, feeding clinic, private practice, early intervention, schools, community health developmental centres, and academia.
van Puymbrouck and Friedman (2024), USA	To explore the clinical experiences and concerns of early‐career occupational therapists entering the workforce during the COVID‐19 pandemic.	Qualitative survey; journal article	3B	<1 year	25%	27	Schools (*n* = 6), acute, outpatient, community, subacute, day rehab, and home health.

Abbreviations: NG, new graduate; NGO, non‐government organisation; NZ, New Zealand; PFD, paediatric feeding disorder; SLP, speech and language pathologist.

^a^ Theses and dissertations are not formally assigned a Level of Evidence or Quality Rating within the Johns Hopkins Hierarchy (Dang et al., 2022).

This scoping review reports the Level of Evidence (LoE) for each included study as ‘… part of the methodological critical appraisal process’ (Brown et al., 2025, p. 11). The LoE hierarchy reflects the rigour of study design (Burns et al., 2011) and supports interpretation of findings (Crowe et al., 2011). The Johns Hopkins Evidence‐Based Practice Model for Nursing and Healthcare Professionals Hierarchy of Evidence (Dang et al., 2022) was used to assign both a LoE (I, experimental; II, quasi‐experimental; III, non‐experimental; IV, expert opinion; V, experiential or non‐research evidence) and a quality rating (A, high quality; B, good quality; C, low quality).

Table 2 (summary of included studies) provides an overview of key information for each included paper, including the assigned levels of evidence and quality ratings. Detailed descriptions of the Johns Hopkins LoE and quality rating system are provided in Table S2.

### 2.5 Stage 5: Collating, summarising, and reporting the findings

Conventional content analysis was chosen as the most appropriate method to analyse the data and synthesise findings (Hsieh & Shannon, 2005). This approach involves deriving codes inductively from the data, without imposing preconceived categories (Hsieh & Shannon, 2005). The research team closely examined the extracted data and met to discuss emerging concepts and generate preliminary codes. All papers were then re‐read in full by the first reviewer (A. B.), who developed the initial coding scheme based on these discussions, highlighting sections that captured key concepts related to the research question. The research team subsequently met again to review and refine the manually derived codes. A. B. then re‐read and re‐coded all papers within NVivo (Lumivero, 2023) to verify, consolidate, and extend the coding structure, ensuring that all relevant data were captured.

Following coding, three members of the research team (A. B., L. C. and P. M.) met and discussed each of the codes, to allow the categories to emerge (Hsieh & Shannon, 2005). In line with the procedure for conventional content analysis described by Hsieh and Shannon (2005), the researchers grouped the codes into the most appropriate categories through a mapping process. These categories were then reviewed collectively, and through discussion on their relationships and shared meanings, four clusters were identified to represent broader patterns within the data. The four clusters are presented in the Section 3.

### 2.6 Positionality

The research team comprises four cisgender, white, able‐bodied academic occupational therapists, with 50+ years combined clinical experience, mainly in paediatric practice. Several team members have direct experience supervising or mentoring early‐career occupational therapists. All team members have also contributed to occupational therapy education and student supervision across university and placement settings. These experiences informed our perspective on factors influencing early career practice. Our backgrounds shaped our approach to this review, and we engaged in regular team discussions to reflect on findings and to support an inclusive review.

## 3 RESULTS

As shown in the PRISMA‐ScR flowchart (see Figure 1), 13 studies met inclusion criteria and were included in the review.

### 3.1 Summary of retained studies

Publication dates for included papers ranged from 1995 to 2024, representing nearly 30 years of research. Papers were predominantly (*n* = 8; 61.5%) published between 2014 and 2024. Seven studies were conducted in Australia, five in the United States, two in New Zealand, and one in Canada. Two studies included participants from two countries. Qualitative (*n* = 7) study design was the most utilised, followed by mixed methods (*n* = 2) and quantitative (*n* = 2) methodologies. Two publications were not studies and were classified as grey literature.

Approximately half of the studies (*n* = 7; 53.8%) focussed on occupational therapists with limited experience following graduation. Participants in these studies were described as new graduates, novice, or early career therapists, with most having fewer than three years of practice experience and some slightly beyond this range to capture relevant early career experiences in paediatric practice. The remaining titles included both less experienced and more experienced therapists in their sample. In seven studies (53.8%), all participants worked exclusively with paediatric caseloads, while in six studies (46.15%), participants worked in mixed caseloads, including both paediatric and adult clients. Importantly, as part of the inclusion criteria, data specific to years since graduation and paediatric practice were distinguishable from other data in these studies. The definition of a ‘new graduate’ occupational therapist varied across the included studies, with some considering clinicians with less than 1 year (*n* = 4), less than 2 years (*n* = 5), and less than 3 years (*n* = 3) of experience as new graduate or novice clinicians. The paediatric practice settings in which new graduate occupational therapists worked most often were school‐based settings (*n* = 9, 69.2%) and ‘general paediatric’ settings (*n* = 7, 53.8%). Participants also reported working in paediatric inpatient settings (*n* = 5, 38.5%), private practice settings (*n* = 5, 38.5%), and early childhood intervention settings (*n* = 1, 7.7%), as new graduate clinicians. Of the papers reporting on mixed practice areas, new graduate participants reported working in mental health, acute care, community, and rehabilitation practice areas. No further clarification regarding the age range of clients within these practice settings was provided.

### 3.2 Level and quality of evidence

Using the Johns Hopkins LoE guide (Dang et al., 2022), three studies (23%) were identified as Level III, Quality A, and eight studies (61.5%) as Level III, Quality B. Non‐research papers (*n* = 2) were not assigned a LoE or quality rating. Overall, three studies received a Quality A rating, indicating high quality according to the Johns Hopkins criteria.

### 3.3 Summary of study findings

The findings within the 13 included papers covered a broad range of topics, as summarised in Table 2. Through content analysis, a total of 393 initial codes were identified, which were then organised into 26 categories and synthesised into four overarching clusters representing key patterns influencing new graduate occupational therapists across paediatric practice. The four distinct clusters identified were (1) preparedness for the role; (2) setting or context matters; (3) support and continuing professional development (CPD); and (4) the new graduate experience. Table 3 presents these clusters along with the number of articles that contributed to each cluster.

**TABLE 3:** Clusters, categories, and codes identified from included articles*.*

Cluster	Category	Code
Preparedness for the role. Graduate‐identified pre‐graduation factors (e.g., university, placements) that supported or hindered effective paediatric practice.	Placement or fieldwork	8
	Preparedness or university curriculum	11
	Reflective practice	6
Setting or context matters. Practice‐setting factors (caseload, resourcing, risk, pressures, role expectations, and team composition) that shape new graduates' experiences in paediatric practice.	Balancing profits of the business	3
	Challenges unique to paediatrics	7
	Clinical caseload	10
	Generalist positions	7
	Scaffolded caseload	2
	Sole occupational therapy	2
	level of clinical risk	4
	Resources	7
	Time and workload pressures	7
Support and CPD. Describes new graduates' views on the support and ongoing training they needed to work effectively in their role, including mentoring, supervision, team support, training, and resourcing.	Access to experienced occupational therapists	10
	Admin support	2
	CPD	11
	Mentoring	12
	Supervision	14
	Team is unsupportive	3
The new graduate experience. Captures new graduates' descriptions of their feelings, confidence, decision‐making, clinical skills, communication, and personal attributes.	Assessment and treatment skills	10
	Difficulty making decisions	4
	Effective communication	2
	Personal attributes (e.g., high expectations or low confidence).	11

### 3.4 Clusters

#### 3.4.1 Preparedness for the role

University curricula and placement experiences emerged as two key areas influencing new graduate preparedness for paediatric occupational therapy practice.

##### University curricula

University curricula were the most frequently discussed contributor to preparedness, with seven papers addressing this topic. Across most studies, new graduates reported a general sense of under‐preparedness from university, which left them feeling overwhelmed and lacking confidence in applying skills and knowledge in paediatric practice settings (Lemire, 2018; Moir, Turpin, & Copley, 2022). Notably, a large national survey by Gray et al. (2012) found that 30% of respondents felt underprepared for paediatric practice upon graduation in Australia. Similarly, in a United States‐based study, (Brandenburger‐Shasby, 2000) found that 14% of surveyed occupational therapists did not feel their pre‐service education had prepared them for school‐based practice. Overall, only 30% of respondents in that study identified that their pre‐service curriculum had adequately prepared them for school‐based practice settings, with specific gaps identified in assistive technology assessment, inclusive service provision, and supporting transitions for children and young people across educational settings (e.g., early childhood to primary school and primary to secondary school) (Brandenburger‐Shasby, 2000). Another specific clinical practice area requiring greater preparation was feeding, with new graduates reporting they felt underprepared to deliver feeding interventions in paediatric practice (Thompson et al., 2024). These findings likely reflect a common experience for new graduates across occupational therapy practice areas, rather than being unique to paediatrics; however, as this review specifically focussed on paediatric contexts, no conclusions can be drawn about other areas of practice.

Importantly, when university content aligned with clinical practice demands, graduates reported a smoother transition to practice (Moir, Turpin, & Copley, 2022). University curricula provided graduates with the basic skills and general knowledge, which served as a reference point for what to do in practice when they felt unsure (Moir, Copley, & Turpin, 2022). In the Brandenburger‐Shasby (2000) study, respondents reported feeling adequately prepared in observation skills, adapting equipment and environments, and school‐specific evaluation approaches. These findings highlight that, while some undergraduate curricula include certain foundational knowledge and skills, more complex or context‐specific aspects of paediatric practice may require greater emphasis to better support graduate preparedness.

##### Clinical placement experiences

A total of six studies shared findings relating to the role of undergraduate clinical placement in preparing new graduate readiness for paediatric practice (Brandenburger‐Shasby, 2000; Lemire, 2018; Moir et al., 2023; Moir, Copley, & Turpin, 2022; Moir, Turpin, & Copley, 2022; Thompson et al., 2024). Placement experiences were consistently identified as a key factor in shaping graduates' confidence and capabilities when transitioning into a paediatric caseload upon graduation. For example, Thompson et al. (2024) found that participants who completed a paediatric feeding placement reported they would not have been prepared to provide feeding intervention upon graduating without this experience. As paediatric feeding was a relatively uncommon topic during their university education, the placement provided an opportunity to build confidence and clinical skills in this area (Thompson et al., 2024). Similarly, participants in papers by Moir, Turpin, and Copley (2022) and Lemire (2018) described feeling ‘thankful’ or ‘lucky’ to have had a paediatric placement, as it assisted in knowing what to do in their current paediatric caseload. Moir, Turpin, and Copley (2022) further identified that student placement experiences supported intervention decision‐making by equipping new graduates with general skills and knowledge from which they could draw. In a later study, Moir et al. (2023) reported that accepting new graduate job offers that are a good match to student placement experiences facilitates a more positive transition from university. Findings from Brandenburger‐Shasby (2000) highlighted the importance of placement experiences in preparing occupational therapists for paediatric roles in schools. In their survey, 35% of respondents indicated placements contributed significantly to their readiness; however, not all respondents completed a school‐based placement (Brandenburger‐Shasby, 2000).

#### 3.4.2 Setting or context matters

Nine studies described the influence of contextual factors on new graduate occupational therapists to deliver effective paediatric service (Clark et al., 2013; Crist et al., 2004; Eberhardt & Mayberry, 1995; Hazelwood et al., 2019; Jackson et al., 2023; Lemire, 2018; Moir et al., 2023; Moir, Copley, & Turpin, 2022; Moir, Turpin, & Copley, 2022). The complexity of paediatric practice, clinical risk, role composition, and workload and organisational demands were the key contextual influences described in the literature.

##### Complexity of paediatric practice

Challenges unique to paediatric practice areas and settings were highlighted by several papers in this cluster. Working across developmental stages, collaborating with multiple stakeholders, and balancing the needs of the child and their family were important factors in achieving engagement from the child in therapy (Jackson et al., 2023; Moir et al., 2023; Moir, Turpin, & Copley, 2022). This complexity is further reflected in the frequent uncertainty about who the ‘client’ is (whether the child, parent or carer, teacher, or school), requiring clinicians to navigate competing priorities (Jackson et al., 2023). Practising in a family‐centred way added further complexity, with new graduates describing how family dynamics and contextual factors impacted on decision‐making (Jackson et al., 2023; Moir et al., 2023; Moir, Turpin, & Copley, 2022). One study described paediatric practice as ‘… extremely complex … there's so many different things then that can impact a child's development and progress. All of the family issues, the things that are going on at school for them, the number of stakeholders. It's never just working with that child’ (Jackson et al., 2023, p. 91, Mentor 5). Similarly, Moir, Turpin, and Copley (2022) reported a new graduate perceived ‘… paediatric decision making as more complex than other areas of their generalist role, due to the variety of factors requiring consideration’ (p. 398).

##### Clinical risk

Clinical risk emerged as a key concern in the literature, with participants in the study by Moir, Turpin, and Copley (2022) sharing that trial‐and‐error approaches were less acceptable in certain paediatric caseloads due to higher clinical risk and where errors could result in poorer outcomes. This awareness of clinical risk prompted graduates to actively seek support for their clinical decision making (Moir et al., 2023). Clark et al. (2013) found that paediatric practice areas, along with vocational rehabilitation and mental health, had the lowest number of near‐misses or reported mistakes. While structured supervision and supportive work environments were noted to influence reporting, the exact reason for these lower rates in paediatric areas of practice was not established.

##### Role composition

The type of caseload, whether generalist or paediatric‐specific, was also seen as enabling or inhibiting new graduate clinicians to effectively work with children and families. Paediatric‐only roles were described as providing greater access to workplace resources, supervision, and informal support. Graduates working in paediatric‐specific practice settings were more likely to have ‘… opportunities to ask questions, seek advice, check their plans, debrief, obtain intervention ideas and reassurance, and solve problems’ (Moir, Turpin, & Copley, 2022, p. 400). Having a caseload with some consistency allowed graduates to draw on previous clinical experiences to assist them in making intervention decisions in situations with similar presentations (Moir, Turpin, & Copley, 2022). In contrast, generalist or sole practitioner roles presented challenges to graduates working with paediatric caseloads, such as fewer resources for their work, feelings of professional isolation, difficulties accessing support (Gray et al., 2012; Hazelwood et al., 2019; Lemire, 2018; Moir et al., 2023; Moir, Turpin, & Copley, 2022), and fewer opportunities to consolidate skills because ‘… pretty much every client you see is completely different’ (Moir, Turpin, & Copley, 2022, p. 401).

##### Workload and organisational demands

Workload expectations and organisational priorities, such as balancing profits of the business, were consistently noted as barriers to effective practice. Participants reported on the pressure experienced by new graduates working in paediatric practice settings to manage the volume of work and fast‐paced environment, and meet billable targets (Jackson et al., 2023; Moir et al., 2023; Moir, Copley, & Turpin, 2022; Moir, Turpin, & Copley, 2022). These demands often reduced the time available to reflect throughout the day and draw on available support mechanisms, particularly in private or profit‐driven settings (Moir et al., 2023; Moir, Copley, & Turpin, 2022). For graduates, such pressures added to the challenge of working in paediatric practice areas and contributed to burnout or leaving their positions (Jackson et al., 2023; Moir, Turpin, & Copley, 2022).

##### Diverse service delivery models

The included studies highlighted a range of practice settings where new graduates worked, including school‐based services, inpatient and outpatient hospital settings, private practices, and early intervention centres. The diversity of these settings influenced the demands on new graduates, affecting caseload composition, workload pressures, and access to resources and support.

#### 3.4.3 Support structures and professional development

Support structures, such as supervision, mentoring and team support, and access to CPD, were presented across nine papers in this review, as enabling new graduate occupational therapists to deliver effective paediatric services. This cluster captures how formal and informal support systems shape the graduate transition from student to therapist.

##### Supervision and mentoring

Supervision and mentoring featured prominently across the extracted data, with clear distinctions evident between the two forms of support. Supervision was reported to assist new graduates with clinical reasoning and intervention decisions specific to paediatric caseloads (Moir et al., 2023; Moir, Copley, & Turpin, 2022; Moir, Turpin, & Copley, 2022). However, barriers to effectively engage in supervision were also discussed. These included a reluctance to seek support from their supervisors due to low self‐confidence or a fear of appearing incompetent, with one participant stating they felt like they ‘… should've learnt that by this point’ (Moir, Turpin, & Copley, 2022, p. 399). One experienced clinician reflected on the potential downside of highly supportive environments, suggesting high levels of support for new graduates may reduce their self‐reliance and impact supervisor productivity over time (Moir, Copley, & Turpin, 2022). In contrast with supervision, mentoring was described more positively, particularly when mentors were external to the organisation or more experienced clinicians (Jackson et al., 2023; Lemire, 2018; Moir et al., 2023; Moir, Turpin, & Copley, 2022; Thompson et al., 2024). One mentee stated, ‘if it's someone external [to the organisation] then you don't feel judgement made about your ability and capacity’ (Jackson et al., 2023, p. 91).

Mentees described mentoring as a non‐judgemental space for new graduates to ask questions, be reassured and validated, and build confidence in particular clinical areas (Jackson et al., 2023; Thompson et al., 2024). Opportunities for job shadowing further supported clinical skill development (Lemire, 2018; Moir et al., 2023; Moir, Copley, & Turpin, 2022). A key barrier to mentorship was access, particularly in sourcing experienced mentors or obtaining employer support for the arrangement (Thompson et al., 2024).

##### Team support and workplace environment

A supportive team was also identified as an important contributor to graduate success in paediatric practice. When graduates worked alongside supportive colleagues, they reported reduced feelings of isolation (Moir, Turpin, & Copley, 2022). Conversely, little workplace support, poor recognition of capabilities, or being a sole clinician contributed to new graduates becoming burnt out (Hazelwood et al., 2019; Moir, Turpin, & Copley, 2022). Supportive practices, such as assistance with caseload management and appointment scheduling, were identified as facilitators of effective practice (Lemire, 2018; Moir, Turpin, & Copley, 2022).

##### Continuing professional development

Access to CPD provided opportunities to further increase skills and knowledge required for paediatric practice (Moir, Copley, & Turpin, 2022). Brandenburger‐Shasby (2000) reported that, within their study of school‐based practice, new graduates perceived a need for an average of five to six in‐service trainings specific to that setting. Thompson et al. (2024) similarly found that CPD was essential for developing competency in assessment and treatment of paediatric feeding disorders. Findings from a school‐based study indicated specific training needs for new graduates, including identifying school‐based intervention techniques, evaluation for assistive technology, developing home/classroom programs, writing appropriate ‘Individualised Education Plan’ goals, and supporting student transitions into the educational system (Brandenburger‐Shasby, 2000). However, common barriers to accessing CPD included financial cost, lack of time, difficulty obtaining leave, and challenges identifying suitable courses (Moir et al., 2023; Moir, Copley, & Turpin, 2022; Moir, Turpin, & Copley, 2022; Thompson et al., 2024).

#### 3.4.4 New graduate experience

This cluster focusses on the personal attributes and clinical capabilities of new graduates as they transition into paediatric occupational therapy roles. It explores the factors that may enable or hinder their ability to navigate the complexities of paediatric practice. Self‐confidence, decision making, communication, and resilience, alongside emerging clinical skills in assessment and intervention, were identified as key factors shaping the new graduate experience.

##### Graduate personal attributes

New graduates often held high self‐expectations and a desire to become experts immediately, which sometimes led to self‐doubt and questioning of their perspectives (Moir et al., 2023; Moir, Turpin, & Copley, 2022). Many new graduates reported feeling ‘out of their depth’ initially, particularly if they had limited personal experience with children (Jackson et al., 2023). A lack of confidence to seek help for clinical decision‐making was also noted (Moir, Turpin, & Copley, 2022). Building resilience, including learning to be flexible, cope with varied pressures, and adapt to challenging situations, was an important aspect of their personal growth (Jackson et al., 2023). Mentoring was frequently raised as a valuable support in fostering this growth, helping graduates grow their confidence and clinical abilities by providing reassurance and validation (Hazelwood et al., 2019; Jackson et al., 2023). A passion for the profession and love of learning motivated proactive seeking of support and ongoing development (Moir, Copley, & Turpin, 2022).

##### Graduate clinical skills

Clinical skills development was a key component of the new graduate experience of working in paediatric practice. Effective communication was highlighted as an important skill for collaborating with parents, teachers, managers, and supervisors to ensure therapy was well‐targeted and aligned with expected outcomes (Lemire, 2018; Moir, Turpin, & Copley, 2022). Decision‐making was frequently described as stressful, overwhelming, and time‐consuming, particularly when making intervention choices (Moir, Turpin, & Copley, 2022). Novice therapists often struggle with knowing how or where to begin treatment (Jackson et al., 2023), commonly using strategies like incorporating children's interests to enhance engagement and continuously monitoring participation to adapt their approaches (Moir, Turpin, & Copley, 2022).

One new graduate identified gaps in their clinical knowledge, particularly regarding sensory processing and its role in achieving occupational outcomes (Lemire, 2018). In United States‐based school settings, new graduates reported frequently using developmental, biomechanical, sensory, and visual motor assessments (Crist et al., 2004). Interventions tended to focus on dressing skills, fine motor coordination, and strength training (Crist et al., 2004). Crist et al. (2004) also reported that new graduates spent most of their time in school‐based roles delivering indirect population‐based services through consultation, with little engagement in system‐level activities like tracking outcomes or evaluating program effectiveness.

Telehealth was discussed as both a facilitator and a barrier to working effectively in paediatric caseloads. It improved family involvement and education by enabling longer and more flexible sessions; however, it also required additional therapist planning time due to limited availability of therapy materials in clients' homes (Van Puymbrouck & Friedman, 2024).

## 4 DISCUSSION

The aim of this scoping review was to identify and synthesise the existing literature on the factors that influence new graduate occupational therapists' ability to work effectively with children and young people aged 0–18 years. From the included studies, four main clusters emerged: preparedness for the role; setting or context matters; support and CPD; and the new graduate experience. The findings provide important insights into how well current education, workplace practices, and professional development support capability growth in new graduate occupational therapists. By mapping these factors, this review contributes to a broader understanding of the professional transition period and highlights priority areas for clinical, educational, and policy‐level interventions. These findings are particularly relevant in the Australian context, where the implementation of the NDIS over the past decade has reshaped service delivery and workforce expectations for new graduate occupational therapists.

A total of 13 studies were included, comprising qualitative, quantitative, and mixed‐methods designs. Using the Johns Hopkins Hierarchy of Evidence (Dang et al., 2022), three studies were rated as Level IIIA (high quality) and eight as Level IIIB (good quality), indicating that, while the studies provide useful insights, the current evidence base remains limited in strength and scope. Notably, no studies examined how clinicians' lived experiences or identities (e.g., gender, culture, or disability) may have influenced their transition to practice or capacity to work within paediatric caseloads. This represents an important gap in the literature, as such factors may also shape new graduates' experiences of entering paediatric roles. Overall, the included papers highlighted relationships between knowledge, support, and environmental contexts in shaping new graduates' capabilities.

Although university curricula and clinical placements provide foundational skills, many new graduates described feeling overwhelmed and underprepared when entering paediatric roles (Moir, Turpin, & Copley, 2022). The variability in both academic and clinical exposure to paediatrics during university education is clear. However, the role paediatric placements play in bridging this gap in paediatric preparation appears important in preparing new graduate occupational therapists to work with children, young people, and families (Brandenburger‐Shasby, 2000). Student placements also had a substantial influence on graduates' sense of preparedness and professional identity, with those whose placements aligned with their first roles reporting smoother transitions, greater confidence, and clearer expectations of their responsibilities (Moir, Turpin, & Copley, 2022).

With preparation varying across graduates, the Paediatric Capability Framework (OTA, 2025b) offers a structured outline of the knowledge, skills, and behaviours expected for occupational therapists across career stages. However, the current review highlights that paediatric practice is shaped by multiple interactive factors that create a complex working environment. Further investigation is needed to understand how these capabilities are operationalised in practice for new graduates, particularly within the realities of variable supervision quality, caseload complexity, service demands, and differing workplace expectations (Murray et al., 2015). Variability in how a ‘new graduate’ was defined across the included studies created uncertainty regarding the duration of the new graduate period, the stage at which foundational capabilities are expected to develop, and the timeframe during which graduate‐specific workplace supports, such as work shadowing or graded caseloads, should be maintained or reduced. Definitions for new graduates in paediatric practice ranged from less than 12 months of experience (Clark et al., 2013; Lemire, 2018; Van Puymbrouck & Friedman, 2024) to 2 years (Eberhardt & Mayberry, 1995; Hazelwood et al., 2019; Moir et al., 2023) or three years post qualification (Brandenburger‐Shasby, 2000; Crist et al., 2004; Gray et al., 2012). This inconsistency in definitions of the new graduate period within paediatric practice highlights the need for clearer, standardised definitions to inform role expectations, supervision requirements, and support structures. Although the Paediatric Capability Framework (OTA, 2025b) provides guidance on paediatric capabilities, it does not address time‐based definitions of new graduates, and further clarification is still needed, particularly regarding what constitutes a ‘non‐complex’ paediatric caseload. Clarifying this terminology would support more consistent approaches to workload allocation and ensure that new graduate occupational therapists receive developmentally appropriate learning opportunities and support.

Within the workplace, effective supervision and mentoring were among the strongest enablers of successful transition to paediatric roles. Graduates described needing access to high‐quality supervision from experienced colleagues, along with informal support to navigate new or complex clinical situations and manage risk (Jackson et al., 2023; Moir et al., 2023; Moir, Turpin, & Copley, 2022). However, access to such supervision was inconsistent, particularly in private practice settings and rural or sole practitioner roles (Moir et al., 2023; Moir, Turpin, & Copley, 2022). Some graduates perceived supervision as evaluative rather than supportive, which may contribute to reluctance to seek guidance when needed (Jackson et al., 2023). Mentoring, particularly from experienced paediatric occupational therapists external to the organisation, was consistently described as beneficial (Jackson et al., 2023; Thompson et al., 2024), providing reassurance, emotional support, and real‐time strategies for clinical decision‐making (Jackson et al., 2023). It remains unclear whether these challenges are unique to paediatric practice, as comparison with other occupational therapy practice areas, such as mental health or aged care, was beyond the scope of this review. Despite the recognised value of mentoring, a clear or consistent framework on how to deliver high‐quality mentoring was not presented in the literature (Moir et al., 2023). These findings support calls for a more structured mentoring framework within paediatric occupational therapy and raise questions about whether mandated supervision for new graduate occupational therapists might offer better development opportunities for those entering paediatric roles, potentially contributing to retention of staff (Jackson et al., 2023).

Graduates' experiences were also shaped by broader workplace factors, particularly the complexity of paediatric practice. Complexity was attributed to diverse clinical presentations, family dynamics, and the shifting developmental needs of the child, especially as they grow (Jackson et al., 2023; Moir et al., 2023; Murray et al., 2015). Graduates were sometimes unclear who the ‘client’ was (the child, parent, teacher, or service) and how to balance competing priorities (Jackson et al., 2023). Rather than suggesting paediatrics is inherently more complex than other practice areas, the findings indicate significant variation in caseload complexity, highlighting the need for clearer scaffolding of caseloads for new graduate clinicians. Although the included studies within this review did not explicitly examine how clinical complexity is managed for new graduates, several papers highlighted the need for graded responsibilities and structured role progression during the transition to practice (Moir, Turpin, & Copley, 2022). Some organisations appear to address this by allocating less complex cases to new graduates initially, then gradually increasing complexity over time as confidence and skills develop. Establishing evidence‐informed guidelines for scaffolding caseload complexity may promote greater consistency and contribute to a safer and more sustainable paediatric workforce.

Alongside complexity, clinical risk was another important influence on new graduates' experiences in this area of practice. The relatively low number of reported near‐misses among new graduate occupational therapists in paediatric settings (Clark et al., 2013) may reflect lower actual risk compared to other practice areas, proactive risk‐mitigation strategies, or underreporting influenced by organisational practices or family involvement in children's care. While the current literature cannot determine which explanation is most plausible, these findings highlight a need for further research examining how new graduates perceive and manage clinical risk within the broader complexity of paediatric practice, and how this shapes their engagement with supervision and self‐directed learning.

Individual characteristics also influence how new graduates experience the transition to paediatric practice. Some new graduates proactively engaged in their own learning, while making effective use of formal supervision and mentoring. Attributes such as resilience, motivation, and a commitment to learning enable these graduates to seek guidance, experiment with strategies, and reflect on their practice (Jackson et al., 2023; Moir, Copley, & Turpin, 2022; Moir, Turpin, & Copley, 2022). However, experienced occupational therapists in one study questioned whether highly supportive environments might inadvertently limit opportunities for developing reflective practice and independent clinical reasoning (Moir et al., 2021). In some contexts, workplace priorities such as billable hours or productivity targets further shape access to high‐quality supervision (Moir et al., 2023). The dual role of supervisor and employer may also influence how comfortable new graduates feel discussing uncertainties or challenges, with some reporting reluctance to seek guidance for fear of appearing incompetent (Moir, Turpin, & Copley, 2022). While these findings highlight common experiences and developmental needs, it is also likely that aspects of identity and personal background shape how challenges are encountered and opportunities accessed, although current literature does not explore these factors in depth.

Finally, CPD was identified as an important facilitator for new graduates working with children and young people (Thompson et al., 2024). However, access to CPD was often limited by time, cost, and availability (Moir et al., 2023). Although some studies identified clinical areas where CPD was particularly valued (e.g., feeding, assistive technology, sensory processing, navigating school‐based systems), these papers were generally older and not specific to the Australian context (Brandenburger‐Shasby, 2000; Thompson et al., 2024). Understanding the current CPD requirements of Australian new graduates and how best to scaffold these across their early years of practice, may support better outcomes for both graduates and the children and families they work with.

### 4.1 Implications and future directions

The findings of this review indicate that the transition into paediatric practice is shaped by intersecting factors related to the individual therapist, workplace, and health system. Several recommendations emerged:

- Clinical practice: Employers should consider caseload complexity, supervision quality, and mentoring when recruiting new graduates. Structured orientation and clear role expectations may ease transitions.
- Education: Ongoing collaboration between universities and paediatric employers may better align curricula and placements with entry‐level expectations. Exploring how capability frameworks are incorporated into curricula could also guide improvements.
- Policy: Consideration should be given to mandated or minimum supervision standards for new graduates in paediatric practice, particularly in private practice settings where access to mentors and structured support is often limited. Additional strategies, such as funded supervision and structured onboarding programs, may further support new graduate occupational therapists and promote a consistent, positive transition into paediatric roles.
- Professional standards and frameworks: Future iterations of capability frameworks, such as the Paediatric Capability Framework (OTA, 2025b), should embed more detailed descriptors of paediatric complexity and scope of practice to provide clearer guidance for new graduates.
- Future research: High‐quality studies are needed to evaluate university curricula, supervision/mentoring models, CPD programs, and caseload designs supporting new graduates. Research should also identify best practices for delivering consistent, high‐quality supervision.

### 4.2 Strengths and limitations

This review used a structured scoping review methodology and included papers published over a 30‐year period, enhancing its comprehensiveness. Limitations include predominance of lower‐level evidence, limited paediatric‐only studies, and varied definitions of ‘new graduate’.

## 5 CONCLUSION

This review identified key skills, attributes, facilitators and barriers affecting new graduate occupational therapists working with children, young people and families. While university education provides foundational knowledge and skills, it may not fully address the complexities encountered in real‐world paediatric settings, particularly those shaped by contemporary Australian disability services, such as the NDIS. Support through manageable caseloads, high‐quality supervision, mentoring, and structured CPD appears key to effective early career development.

The Paediatric Capability Framework (OTA, 2025b) provides a useful foundation for defining expected competencies. However, greater clarity is needed in operationalising these capabilities to support new graduates in their early years of paediatric practice. Future research should examine how paediatric workplace practices can better support transitions from student to professional, considering supervision quality, caseload complexity, and ongoing learning opportunities. Developing a sustainable and capable paediatric workforce will rely on aligning education, practice, and policy to the real‐world experiences of new graduates.

## AUTHOR CONTRIBUTIONS

All authors contributed to the conceptualisation, methodology, formal analysis, and interpretation of the findings. The first author was responsible for project administration, data curation, and drafting the original manuscript. All authors contributed to reviewing and editing the manuscript and approved the final version.

## ETHICS STATEMENT

Not applicable.

## CONFLICT OF INTEREST STATEMENT

The authors have no conflict of interest to declare.

## DECLARATION OF GENERATIVE AI AND AI‐ASSISTED TECHNOLOGIES

No generative artificial intelligence tools were used in the preparation or writing of this manuscript. All content was written, reviewed, and approved by the authors.

## Supporting information


**Table S1:** Population, Concept, and Context Framework.
Table S2: Johns Hopkins Levels of Evidence and Quality Ratings Guide.

## Data Availability

No new data were created or analysed in this study rendering data sharing not applicable to this paper.
