# Supplementary material for: Exploring facilitators and barriers to effective practice among new graduate occupational therapists working with children and families: A scoping review
Source: Aust Occup Ther J. 2026 Apr 27;73(3):e70092. doi: 10.1111/1440-1630.70092 (PMC13112326; doi:10.1111/1440-1630.70092)
Supplement: Supplementary file 1 — Table S1: Population, Concept, and Context Framework. Table S2: Johns Hopkins Levels of Evidence and Quality Ratings Guide. [file AOT-73-0-s001.docx]

**Supplementary Table S1:** Population, Concept, and Context Framework

| **Component** | **Description** |
| --- | --- |
| **Population** | New graduate occupational therapists working with children aged 0-18 years. Studies were included if participants had fewer than three years of experience, regardless of whether they were specifically labelled as ‘new graduates’ or ‘novice’. Where participant experience or practice setting was not clearly described or slightly exceeded 3 years of experience, inclusion was determined by examining the results or discussion section for relevant details. |
| **Concept** | Experiences, roles, skills, and capabilities of new graduate occupational therapists in paediatric practice. This included strategies, facilitators, barriers, and challenges related to practice with children. |
| **Context** | Any paediatric practice area was considered, including practice settings such as schools, hospitals, community, or private practices. Studies from all geographic locations and funding models were included, due to limited available literature. No restrictions were placed on practice area, practice setting or geographical location. |

**Supplementary Table S2: Johns Hopkins Levels of Evidence and Quality Ratings Guide**

| **Level** | **Type of Evidence** | **Quality Rating** | **Description** |
| --- | --- | --- | --- |
| I | Experimental study, Randomised Controlled Trial (RCT), or meta-analysis of RCTs | A High | Consistent results with sufficient sample size; definitive conclusions; comprehensive literature review with thorough reference to scientific evidence. |
|  |  | B Good | Reasonably consistent results; sufficient sample size; fairly definitive conclusions; fairly comprehensive literature review with some reference to scientific evidence. |
|  |  | C Low | Little evidence with inconsistent results; insufficient sample size; conclusions cannot be drawn. |
| II | Quasi-experimental study | A High | Consistent results with sufficient sample size; definitive conclusions; comprehensive literature review with thorough reference to scientific evidence. |
|  |  | B Good | Reasonably consistent results; sufficient sample size; fairly definitive conclusions; fairly comprehensive literature review with some reference to scientific evidence. |
|  |  | C Low | Little evidence with inconsistent results; insufficient sample size; conclusions cannot be drawn. |
| III | Non-experimental study, qualitative study, or meta-synthesis | A High | Clear aims and objectives; consistent methodology; conclusions supported by data. |
|  |  | B Good | Aims and objectives are stated; methodology is consistent; conclusions supported by data. |
|  |  | C Low | Aims and objectives not stated; methodology is inconsistent; conclusions not supported by data. |
| IV | Opinion of nationally recognised experts based on research evidence or consensus panels | A High | Expertise is clearly evident; recommendations are based on evidence that is clearly referenced and systematically developed. |
|  |  | B Good | Expertise is evident; recommendations are based on evidence that is referenced but not systematically developed. |
|  |  | C Low | Expertise is not evident; recommendations are not based on evidence. |
| V | Literature reviews, quality improvement, program or financial evaluation, case reports, opinion of experts | A High | Clear aims and objectives; consistent methodology; conclusions supported by data. |
|  |  | B Good | Aims and objectives are stated; methodology is consistent; conclusions supported by data. |
|  |  | C Low | Aims and objectives not stated; methodology is inconsistent; conclusions not supported by data. |
